# Nanoemulsions for “Nose-to-Brain” Drug Delivery

**DOI:** 10.3390/pharmaceutics11020084

**Published:** 2019-02-17

**Authors:** Maria Cristina Bonferoni, Silvia Rossi, Giuseppina Sandri, Franca Ferrari, Elisabetta Gavini, Giovanna Rassu, Paolo Giunchedi

**Affiliations:** 1 Department of Drug Sciences, University of Pavia, Pavia 27100, Italy; silvia.rossi@unipv.it (S.R.); giuseppina.sandri@unipv.it (G.S.); franca.ferrari@unipv.it (F.F.); 2 Department of Chemistry and Pharmacy, University of Sassari, Sassari 07100, Italy; eligav@uniss.it (E.G.); grassu@uniss.it (G.R.); pgiunc@uniss.it (P.G.)

**Keywords:** blood-brain barrier, brain targeting, nanoemulsion, nose-to-brain delivery, nasal mucosa, olfactory pathway

## Abstract

The blood–brain barrier (BBB) plays a fundamental role in protecting the brain from toxic substances and therefore also controls and restricts the entry of therapeutic agents. The nasal administration of drugs using the nose-to-brain pathway allows direct drug targeting into the brain, avoiding the first-pass effect and bypassing the BBB. Through the nasal route, the drug can access the brain directly along the trigeminal and olfactory nerves, which are located in the upper part of the nasal cavity. Nanoemulsions are formulations belonging to the field of nanomedicine. They consist of emulsions (commonly oil in water) stabilized by one or more surfactants—and eventually co-surfactants—delivered in droplets of small dimensions (sizes of 100–300 nm or less) with a high surface area. A mucoadhesive polymer such as chitosan can be added to the formulation to impair rapid nasal clearance. Nanoemulsions represent promising formulations to deliver drugs directly into the brain through the intranasal route. Therefore, they can be used as a possible alternative to oral administration, avoiding problems such as low solubility in water, poor bioavailability, enzymatic degradation and slow onset of action. This review focuses the present situation in literature regarding the use of nanoemulsions for nose-to-brain targeting, with particular attention to recent publications. Nasal nanoemulsions appear to be effective, non-invasive and safe drug delivery systems to achieve brain targeting for the treatment of neurological diseases.

## 1. Introduction

The microvasculature of the central nervous system (CNS) is defined as a blood–brain barrier (BBB) because it isolates the brain from the remaining part of the body. CNS vessels, at the levels of arteriole–capillary–venules, are non-fenestrated and continuous; they are able to control the exchange of molecules, ions and cells between the blood and the brain [1]. This strong barrier capacity allows the BBB to protect the CNS from pathogens, toxins and external agents.

The barrier structure of BBB depends on the specific properties of the brain endothelial cells (BECs) that constitute the walls of the blood vessels. BECs are different from endothelial cells of non-neural tissues—they are polarized cells, held together by tight junctions that strongly limit the paracellular flux of solutes (molecules and ions) and restrict vesicle-mediated transcellular transports (pinocytosis/transcytosis) [1,2]. BECs are characterized by two kinds of systems of transport in both directions that include the expression of efflux transporters, whose function is the elimination of the lipophilic toxins able to passively diffuse through the cell membrane, and the expression of influx transporters, which are specific carriers that deliver the nutrients across the BBB into the brain [1]. Among efflux transporters, P-glycoprotein plays an important role, as its activity has been associated with diseases of the CNS such as drug-resistant tumors and epilepsy [1].

BBB is not only a cellular self-defense barrier but also an active interface that strictly controls the CNS microenvironment, allowing its normal neuronal functions. BBB cells are able to communicate with CNS cells and they adapt their behavior to the needs of the CNS, responding to pathological conditions and sometimes becoming a cause of the progression of the disease [1]. 

Comprehension of the mechanisms that regulate the BBB during health and their modification during disease involve interdisciplinary studies that several researchers have put correctly into evidence and can give an important support to finding the correct therapeutic treatments for a wide series of neurological disorders [3,4].

Neurological diseases, such as neuroinfections, Parkinson’s disease, Alzheimer’s disease, multiple sclerosis, chronic age-related neurodegenerative diseases, cerebral ischemia and so on represent a group of severe pathologies with a broad spectrum of pathological conditions that result in alterations of neural functions. Healthcare statistics confirm that the incidence of CNS diseases is rapidly increasing around the world with higher healthcare costs [5].

The current therapies have a significant role in the treatment of CNS diseases, resulting in increased survival rates, but they still present unsolved problems, and complete cures are lacking for most CNS diseases, so therapeutic advances are needed. The main problem is that the treatment of CNS diseases requires therapeutics that are able to cross over the BBB in sufficient quantities to achieve the necessary therapeutic levels. However, the delivery of drugs to the brain is a big challenge as the restrictive nature of the BBB represents a remarkable obstacle for drug delivery to the CNS. It is estimated that the permeation of the barrier towards the CNS does not occur for about 98% of active substances of low molecular weight and for nearly 100% of macromolecules (Pardridge 2005) leading to a dramatically low bioavailability in the target site.

For a direct delivery to brain, the drug can be administered through local delivery strategies such as intra-cerebroventricular or intra-parenchymal injections, intracranial delivery with mini-pumps, catheter infusions, focused ultrasound approaches or external electromagnetic field-based methodologies. However, all of these techniques are very invasive and risky, in particular because of the need for surgical intervention [6], and many of them are not suitable in cases of multiple or chronic regimens. 

For these reasons, many efforts have been made to design strategies to bypass the BBB for the delivery of active substances to the target site. The design of a strategy for brain targeting means to use non-conventional administration routes and, in turn, to design drug formulations with properties suitable for optimal delivery through these routes.

The nose is responsible both for respiration and for olfaction. The human olfactory region, where olfactory and trigeminal nerve terminations are present, occupies 2–12.5 cm^2^, representing approximately 1.25–10% of the total surface area of the nasal cavity, and it is about 60 μm thick [7]. Olfactory and trigeminal pathways are the only routes by which the brain is connected to the outside environment [8,9]. Thanks to the direct connection provided by the olfactory and trigeminal nerves present between the olfactory epithelium and the brain, drug targeting can be achieved with the administration of formulations onto nasal mucosa [10,11,12]. Therefore, particular attention must be given to studies of the blood–nerve barrier (BNB), which consists of endoneurial microvessels within the nerve fascicle and the investing perineurium [13]. These microvessels are actively involved in the mechanisms that regulate the permeability of the perineurium and endoneurial capillaries, and surely they play an important role in the passage of substances from olfactory and trigeminal pathways into the CNS.

Nose-to-brain drug delivery is a painless, non-invasive administration route that can be used to deliver therapeutic agents into the brain by bypassing the BBB [10,14,15,16,17].

These drug administration pathways are characterized by many advantages such as increased patient compliance, high safety, remarkable ease of administration and rapid onset of action, as well as minimized systemic exposure [7]. Furthermore, the use of nasal mucosa as a route of drug administration permits drugs to avoid hepatic first-pass metabolism. Consequently, nasal doses are often 2–10 times lower than oral doses.

Direct transport of drug to brain through nasal administration is therefore more promising than oral or intravenous routes of administration [18].

**Figure 1:**
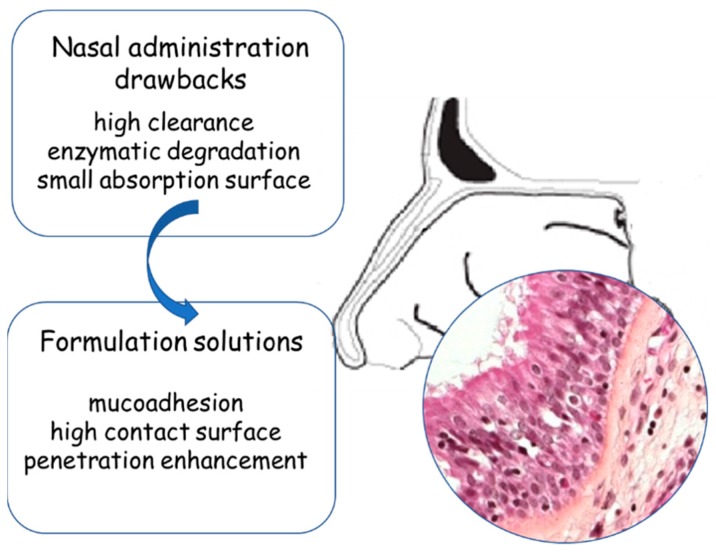
The nose as a route for the administration of drugs.

However, despite its numerous advantages, nose-to-brain drug delivery can be limited by possible low bioavailability due to enzymatic degradations of sensitive drugs onto the mucosal surface, high clearance and restrictions determined by the anatomy of the nasal cavity (e.g., small volume, limited surface area of the olfactory mucosa, mucociliary clearance, etc.). These problems should be correctly addressed in designing suitable nose-to-brain formulations (Figure 1). Despite these limitations, examples of promising results are present in clinical trials [19,20].

According to the present literature, different kinds of nanocarriers are used to prepare nasal formulations able to target the brain, constituted by polymer-based and lipid-based nanoparticles [21,22,23]. Among nanocarriers, the liquid dispersed systems represented by nanoemulsions (NEs) are attracting more and more interest in nose-to-brain delivery. 

The aim of this review is to define the present situation, according to literature, regarding the use of NEs for the treatment of neurological pathologies through the nose-to-brain route.

## 2. General Characteristics of NEs in Brief

Nanoemulsions (NEs) are oil-in-water (O/W) or water-in-oil (W/O) dispersions of two immiscible liquids stabilized using appropriate surfactant(s) [24], with a mean droplet diameter of about 100 nm [25], even if in literature upper size limits up to 300 nm [26] have been reported. As the size of the droplets is significantly smaller than the wavelength of visible light, NEs have either a transparent or from transparent-to-milky-white appearance [25].

NEs can be formulated into different kinds of dosage forms like liquids, creams, gels, foams, sprays and so on, and can be administered by different routes including oral, parenteral and ocular, in addition to nasal [27].

NEs have droplets of a small size and therefore they are characterized by a higher surface area with respect to other formulations, and by a long-term physical stability, because the small droplet size impairs destabilization phenomena like coalescence, creaming and sedimentation. 

NEs can be used to solve problems of drug solubility and/or of drug stability (oxidation, pH, hydrolysis and enzymatic degradation at the mucosal level, under physiological conditions) [24,28]. Hydrophobic drugs are expected to dissolve in the oily phase, and when the drug (dissolved in the oily phase) is released from the NE and comes in contact with the surrounding aqueous environment, a nanoprecipitation can occur. This determines the formation of particles with an enormously high surface and a remarkable improvement of drug dissolution rate, according to the Noyes–Whitney equation [29]. NEs can be used also to mask the bitter or unpleasant taste of drugs [29] and to carry products of natural origin [30,31].

**Figure 2:**
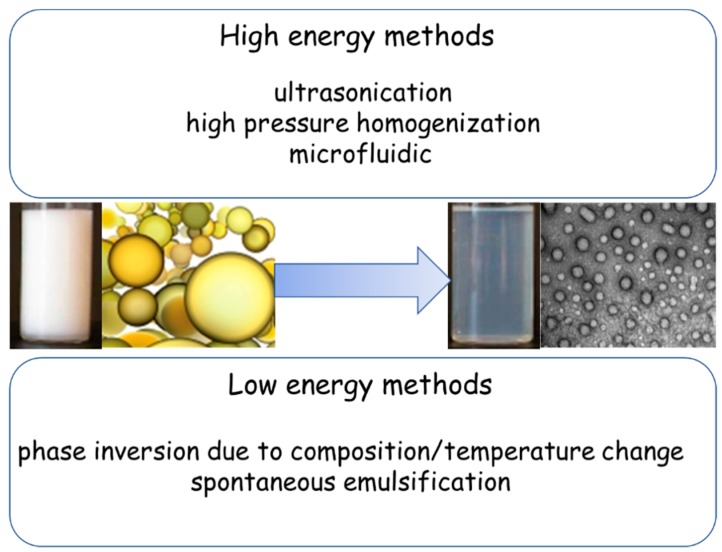
Preparation methods of nanoemulsions (NEs).

The preparation methods of NEs have been reviewed in exhaustive papers [24,32]. According to these authors, NEs can be prepared through different techniques that can be classified into two broad categories: high-energy methods and low-energy methods (Figure 2). In the case of high-energy methods, such as ultrasonication and high pressure homogenization, the constitution of the small droplets involves a mechanical device that generates disruptive forces breaking up oil and water phases to produce small oil droplets, a process that consumes significant energy. The devices used are microfluidic, ultrasound or high pressure homogenizers [33].

The low-energy methods involve specific physico-chemical processes such as phase inversion temperature and emulsion inversion points to make small droplets without consuming significant energy. In the low-energy methods, the droplets are constituted when the system undergoes an inversion of phase in response to changes, such as composition or temperature, and consequently passes through a low interfacial tension state [32].

The quantity of lipidic component(s) used in NEs is dependent on the kind of emulsion (generally they contain 5–20% lipidic droplets in cases of O/W emulsion) and on solubility of the drug to loaded in the system [29]. The solubilizing capacity of the oil phase is a factor that plays an important role in the oil selection because the oil phase must dissolve and maintain dissolved the drug. Furthermore, the highest possible solubility is required to decrease the amount of oil used, reducing the required amounts of surfactants that might determine toxicity problems [34].

Usually the lipids used for the preparation of NEs, alone or in mixture, are fractions of oils of natural origin such as sesame oil, cottonseed oil, soybean oil, coconut oil, and so on that can be classified according to chain lengths as long-chain, medium-chain and short-chain triglycerides [29].

The kind of oil components used in the formulation process of NEs influences (sometimes strongly) the bioavailability of the drug. Many studies have been performed to clarify this aspect for oral NE delivery. For example, it is reported that the bioavailability of curcumin is maximized in NEs made with medium-chain and long-chain triglycerides [35]. Furthermore, it has been reported that several NEs are characterized by a direct lymphatic absorption (avoiding first-pass metabolism) [29] and it is likely that this behavior strongly depends on lipidic phase composition. No data seem available at the moment regarding the relevance of oil choice for nasal and in particular for nose-to-brain administration.

Emulsifiers are needed to facilitate NE formation and to ensure its kinetic stability during storage, even if NEs typically require less surfactant than other colloidal dispersions. The emulsifier used for the preparation of NEs is very often a surfactant, but proteins and lipids have also been used. Lecithin (phosphatidylcholine) [36], sodium deoxycholate (bile salt) [37], polyoxyethlene sorbitan monolaurate (Tween 20, 40, 60, 80) [38] and sorbitan monolaurate (Span 20, 40, 60, 80) [39] are commonly employed in the preparation of NEs. Other surfactants proposed are poloxamers [40], sodium dodecyl sulfate [41], amphiphilic proteins like casein [42], polysaccharides (starch derivatives, gums) [43] and poly-ethylene-glycol (PEG)-containing block copolymers [44].

Co-surfactants, such as polyethylene glycol, ethylene glycol, propylene glycol, ethanol and glycerine can be used for the stabilization of NEs [45]. All of these can be used alone or in combination. The selection of the surfactants is critical, as they must be safe at the concentrations used.

The choice of the surfactant or of the surfactant blend influences both the size of the droplets and the stability of the NE. However, it must be taken into account that this choice can influence pharmacokinetics and toxicity; for example, poloxamer 188 has renal toxicity at a concentration higher than 0.5% in parenteral NEs [29].

Generally speaking, these formulations appear safe because, to our knowledge, no mutagenic effects are reported in literature, and only safe pharmaceutical excipients have been used for their preparation.

In the present literature there is some confusion regarding the classification of NEs because they are sometimes confused with microemulsions, which are thermodynamically stable systems that form spontaneously [24,46]. For this reason, macroemulsions and NEs can be prepared with high- and low-energy methods, while microemulsions are prepared with low-energy methods only. Important differences can be found between classical macroemulsions (classical emulsions), NEs and microemulsions in droplet size range and stability characteristics [32,47].

## 3. General Overview of NEs for Nose-to-Brain Delivery

The easiest classification of NEs designed for nose-to-brain delivery is based on the drug loaded and the therapeutic purpose. As the final target is the brain through the nose, the pharmacological actions regard pathologies of the CNS. In one case, a probe is loaded in the NE to obtain brain imaging. The formulations used in the intranasal administrations of drugs are always, in our knowledge, O/W emulsions. 

A general overview of the present literature about NEs for nose-to-brain targeting shows clearly that intranasal use is often an alternative to the oral therapy. In fact, if the drug is administered orally to reach the brain, this kind of administration can present problems for some drugs, which are summarized in Table 1. CNS delivery through the nasal mucosa sometimes performs better than parenteral administration as well, as shown by in vivo experiments.

One of the first examples in the literature of the use of NEs to reach the brain through the administration onto nasal mucosa is a paper by Kumar et al. in which NEs were utilized to carry risperidone, an antipsychotic drug belonging to the group of benzisoxazole derivatives [48]. This drug is available in trade as oral formulations (tablets and oral solutions) which are characterized by a problem of low bioavailability, mainly owing to the first-pass hepatic metabolism. Furthermore, the systemic oral administration has many side effects. Risperidone NEs were prepared using Capmul MCM as the oily phase (8%, *w*/*w*) and Tween 80 as a surfactant. Risperidone mucoadhesive NE was prepared adding chitosan (0.50%, *w*/*w*) to NEs and stirring the dispersion for 1 h. In vivo studies were carried out on Swiss albino rats: Drug distribution in the blood and in the brain following intranasal and intravenous administrations of NEs and risperidone solutions were determined using technetium (99mTc) labeling. These studies showed more rapid and larger drug transport into the CNS after the intranasal administration of the mucoadhesive chitosan-containing NEs in comparison with nasal and intravenous NEs and solutions. Analogous results were obtained by the same authors preparing drug-loaded NEs containing olanzapine, a second-generation antipsychotic agent with broad efficacy [49]. According to the authors’ opinion, these positive results were related to the enhancing of the nasal retention time due to the presence of chitosan, thus confirming the importance of this polymer as mucoadhesive agent in nasal formulations [50].

Yu et al. prepared a formulation that they defined as “submicron emulsion”, containing an antiaging compound, ergoloid mesylate, which is constituted by methanesulfonate salts of the three alkaloids dihydroergocristine, dihydroergocornine and dihydroergocryptine [51]. Egg lecithin was used as the main emulsifier. In vivo studies were carried out on male Sprague–Dawley rats: Nasal administrations of ergoloid mesylate “submicron emulsions” were compared to nasal and intravenous administrations of drug solutions. The Area Under the Curve (AUC) and the absolute bioavailability in the cerebrospinal fluid (CSF) following intranasal administration of the “submicron emulsions” were higher than those obtained after nasal administration of the solutions.

These promising results were assumed as an example for further studies involving drugs for the therapy of other CNS pathologies. Antiepileptic intranasal drug-loaded mucoadhesive NEs loaded with amiloride for nose-to-brain delivery were prepared by Jain et al. [52]. Bahadur et al. prepared NEs loaded with ziprasidone hydrochloride, an antipsychotic drug [53]. NEs with mucoadhesive properties were obtained through the addition of chitosan in the formulations. Nasal ciliotoxicity studies revealed that the formulations containing ziprasidone were free from acute toxicity, and pharmacodynamic studies showed good results in locomotor activity tests and paw tests. 

An important application of nose-to-brain delivery with NEs was that described by Mahajan et al., who used NEs to carry anti-HIV drugs [28]. It is known that after the initial infection, CNS is the region in which HIV viruses constitute a sort of “anatomic reservoir”, and from which they can reactivate the infection. Furthermore, the brain infection from HIV can determine neuro-AIDS, a form of dementia and cognitive impairment. It is clear that improved drug delivery to the CNS will reduce the possibility of underlying persisting infections. Saquinavir mesylate is a protease inhibitor with activity against HIV-Type 1 (HIV-1). However, its bioavailability is low, owing to its low solubility in water. Furthermore, saquinavir permeability through the BBB is poor and is a P-glycoprotein and cytochrome P450 substrate. For all these reasons, nasal O/W NEs containing saquinavir mesylate were prepared by the spontaneous emulsification technique using Capmul MCM, a mono-diglyceride of medium-chain fatty acids (mainly caprylic and caproic). NEs were characterized in terms of drug content, droplet size and zeta potential. Ex vivo permeation studies were carried out using excised fresh sheep nasal mucosa. NEs showed an increase in drug permeation compared to plain drug suspension. Cilia toxicity was low. In vivo biodistribution studies, carried out after nasal administration of 99mTc formulations, showed higher drug concentration in the brain after nasal administration of NE with respect to intravenous administration. Gamma scintigraphy imaging of a rat brain demonstrated increased drug transport to the CNS after NE nasal administration.

Pathak et al. prepared nimodipine-loaded NEs for the treatment of senile dementia and cerebrovascular spasms [54]. Nimodipine is a dihydropyridine calcium channel blocker used in oral treatments, but it has several problems, including poor oral bioavailability (5–10%) due to low water solubility and first-pass metabolization, and consequently low brain concentration of the drug. Furthermore, nimodipine is a P-glycoprotein substrate. The authors in fact put into evidence that P-glycoprotein-mediated drug efflux can be one of the causes of low brain drug levels. The aim of the work was the development of in situ gelling mucoadhesive NEs for nasal delivery of nimodipine. In the study, different polymers (Carbopol 934 P, chitosan, sodium alginate and sodium CMC) were tested as mucoadhesive agents. Pluronic F 127 and Pluronic F 68 were screened as gelling agents. The drug was dissolved in Capmul MCM as oil, using Labrasol as surfactant, and Transcutol P as co-surfactant. Pseudoternary phase diagrams of oil, surfactant, co-surfactant and water were made for the development of the formulations using a water titration method to define the optimal concentrations of components. Ex vivo drug permeation studies were carried out using freshly excised goat nasal mucosa and showed no toxicity on the mucosa. In vivo studies carried out in rats demonstrated high plasma and brain concentrations of nimodipine in the case of NE formulations containing Carbopol 934 P. The authors concluded that this formulation strategy may also be effective for targeting other therapeutic entities to the CNS with low bioavailability and brain uptake.

NEs have also demonstrated the ability carry active principles of natural origin, and NEs loaded with curcumin led to formulations with interesting potentialities [55]. Curcumin is a phenolic phytochemical achieved from the rhizome of *Curcuma longa* L. The curcumin oral administration in Alzheimer’s disease animal models determines the inhibition of Amiloid beta (Aβ) peptide oligomerization and deposition in the brain [56]. Furthermore, curcumin has been found to improve memory and cognitive deficits in rats [57]. However, the efficacy of this drug is limited by its low aqueous solubility, poor absorption from the gastrointestinal tract and rapid metabolism. For these reasons, a study was carried out about the development of curcumin-loaded NEs for intranasal delivery to the CNS [55]. NEs were prepared using the spontaneous nanoemulsification method, adding curcumin to the oil phase (Capmul MCM). Chitosan was added to obtain mucoadhesive NEs. The goal of the study was to optimize the curcumin NE formulation process using a Box–Behnken design that was constructed using oil, surfactant and co-surfactant concentrations as independent variables. Globule size and zeta potential were studied as responses. The concentrations of oil and surfactant were found to be critical for obtaining the desired globule sizes, whereas the addition of chitosan affected zeta potential of NEs. In vitro cytotoxicity studies were carried out using SK-N-SH cell line, showing that the formulations determined no toxicity. Ex vivo diffusion studies were carried out with Franz diffusion cells: Chitosan-containing NEs showed the highest flux and permeation across the mucosa compared to NEs without chitosan and drug solutions, confirming the importance of chitosan not only as mucoadhesive polymer but also for its penetration enhancing properties.

O/W NEs containing resveratrol were prepared by Pangeni et al. [58]. Resveratrol is the most biologically active compound present in grapes and red wine, and known for its properties of reducing the production of amyloid peptides, in addition to its cytoprotective actions and reduction of cognitive defects [59]. NEs were prepared through the spontaneous emulsification method, followed by high pressure homogenization. Vitamin E and Sefsol (1:1) were used as oil phase (Sefsol is a propylene glycol mono caprylic ester), Tween 80 as surfactant and Transcutol P as co-surfactant. Significantly high ex vivo trans-nasal mucosal fluxes were found using a Franz diffusion cell on porcine nasal mucosa. Pharmacokinetic and brain-targeting studies carried out on Wistar rats demonstrated a higher concentration of the drug in the brain after nasal administration of NEs. Furthermore, histopathological studies showed decreased degenerative changes in the case of resveratrol NE administration.

In another research work, Nasr studied the co-encapsulation of the two polyphenols, resveratrol and curcumin (1:1 weight ratio) in mucoadhesive NEs based on hyaluronic acid for nose-to-brain targeting [60]. For the preparation of NEs, the lipidic phase was constituted by Labrafac lipophile and Labrafac PG, while the surfactants were Tween 80 and Cremophor RH 40. NEs were prepared in the dark to avoid resveratrol conversion from the trans to the cis isomer that can occur in solution because of its photosensitivity. This work is a good example of the use of NEs as formulations that are able to protect loaded drugs from degradation and preserve their antioxidant properties. Furthermore, this work demonstrates the capability of NEs for loading two drugs together. Ex vivo flux across freshly isolated sheep nasal mucosa was studied, and diffusion of the two drugs was achievable for 6 hours. In vivo quantification of the two drugs in rat brains showed an increase of the amounts of the two polyphenols in the brain with respect to the administration of solutions.

Yadav et al. compared pharmacokinetics and bioavailability studies of NEs containing cyclosporine-A when administered by nasal and intravenous routes in Sprague−Dawley rats [61]. Cyclosporine-A is an 11 amino acid peptide. It has been reported that it can have potential neuroprotective effects that can be achieved only with very high oral doses. However, such high doses determine negative side effects, such as immune suppression, hepatotoxicity and nephrotoxicity. For these reasons the use of orally administered cyclosporine-A as neurotherapeutic has not been considered so far. This study demonstrated that cyclosporine-A is not efficiently transported into the brain through the BBB upon intravenous administration, while nasal administration of cyclosporine-A loaded NEs is an effective way of brain targeting. The nasal administration of NEs enhances the brain concentration of cyclosporine-A and significantly limits its peripheral exposure and toxicity. 

The same authors evaluated the therapeutic use of nasal cationic NEs encapsulating an anti- tumor necrosis factor-alpha (TNFα) siRNA, for potential anti-inflammatory therapy, as neuroinflammation is often seen in patients with neurodegenerative disorders [62]. The study has shown that intranasal delivery of TNFα siRNA NEs in rats led to a higher uptake of siRNA in the brain compared to distribution into the blood.

NEs loaded with riluzole, a drug approved for the treatment of amyotrophic lateral sclerosis (ALS), have been recently proposed [63]. ALS is a progressive neurodegenerative disorder belonging to the group of motor neuron diseases and characterized by the progressive deterioration of upper and lower motor neurons. Riluzole is a drug belonging to Biopharmaceutical Classification System (BCS) class II that has 60% absolute bioavailability, also because it is a substrate of P-glycoprotein. Furthermore, it is not able to cross the BBB and to reach the brain. Riluzole-loaded NEs were prepared with a drop size of about 24 nm, and and the results were free from nasal ciliotoxicity and stable for three months. Drug brain uptake following the intranasal administration of riluzole NEs was significantly higher with respect to the oral administration of the same NEs. The conclusion was that intranasal administration is a potential approach for ALS treatments.

Selegiline, a Monoamine oxidase B (MAO-B) inhibitor with neuroprotective and antioxidant effects, is utilized for the oral therapy of Parkinson’s disease, Alzheimer’s disease, depression, narcolepsy and cocaine addiction [64]. Recently, some neurologists have proposed selegiline monotherapy for patients that are in the early stage of Parkinson’s disease [64]. This allowed to delay the treatment with l-dopa, thus avoiding l-dopa side-effects for a certain period. The problems connected with the oral administration of this drug are the low drug bioavailability (10%) due to its poor solubility in water and a remarkable first-pass metabolism. Furthermore, low amounts of selegiline reach the brain owing to the presence of the BBB, which restricts the entry of the drug into the brain both by oral and intravenous administrations. Kumar et al. have proposed NE loaded with selegiline for direct nose-to-brain delivery [65]: The selection of excipients was done on the basis of solubility and miscibility studies carried out using different oils. Selegiline showed highest solubility in a mixture of grape seed oil and Sefsol 218 (1:1). Grape seed oil consists of unsaturated fatty acids and contains tocopherols, phytosterols and omega-3 fatty acids. Omega-3 fatty acids are known to be modulators of neuronal functions and have a role in oxidative stress-mediated pathways in the CNS. Therefore, grape seed oil has a synergistic antioxidant activity with selegiline. Among surfactants, selegiline showed highest solubility in Tween 80, which was used for the preparation of the leader formulation. Ex vivo permeation studies through porcine nasal mucosa were carried out on segeline-loaded NE, using a drug suspension as a comparison. The studies showed that NE was characterized by a high flux, which was very low in the case of the suspension. The authors affirmed that the low permeation rate of drug suspension with respect to the NE could be due to the fact that selegiline is a substrate of P-glycoprotein, meaning that it could not diffuse across the nasal epithelial membrane due to the presence of a P-glycoprotein efflux pump. In the presence of Tween 80, a P-glycoprotein inhibitor, the efflux pump activity was reduced. The efficacy of the leader formulation was tested for Parkinson’s disease using behavioral studies, specifically with Wistar rats. The results of behavior studies showed that treatment of haloperidol-induced Parkinson’s disease in rats with administered intranasal selegiline NEs was characterized by a remarkable improvement in behavioral activities in comparison to the oral administrations.

Another interesting application of nose-to-brain delivery of NEs is in the therapy of cerebral ischemia, which is a severe disease that represents a leading cause of death and disability in the aged population. Oxidative stress is one of the primary factors that exacerbates the damage of cerebral ischemia [66]. It has been reported that thymoquinone, one of the constituents of the volatile oil from *Nigella sativa* seeds, has antioxidant properties and that could have a potentially positive role in the treatment of cerebral ischemia [67]. However, thymoquinone has poor bioavailability owing to its low water solubility, extensive metabolism and rapid elimination. Thymoquinone mucoadhesive NEs were prepared by the ionic gelation method using oleic acid as oil, carbitol as co-surfactant and Tween 20/labrasol/cremophore EL as surfactants; the continuous phase was purified water [68]. Mucoadhesive NEs were prepared through addition of a mucoadhesive polymer (chitosan). The NEs had a mean globule size of about 100 nm. Ex vivo permeation studies across goat nasal mucosa were performed showing that the incorporation of chitosan increased the permeability of the NEs, confirming the penetration enhancing properties of this polymer due to its transient opening of tight junctions. Wistar rats (with middle cerebral artery occlusion-induced focal cerebral ischemia) were used for in vivo studies, and showed enhanced drug bioavailability in the brain after nasal administration as compared to intravenous administrations.

Recently, an interesting study has been carried out by Pandey et al. [69] regarding an application of nasal administration of NEs for the treatment of depression, a disease that will rise in the near future, according to the World Health Organization’s predictions. Paroxetine is a selective serotonin inhibitor used for the treatment of depression and anxiety. When administered orally this drug has poor bioavailability (less than 50%) due to the hepatic first-pass effect. Furthermore, the BBB restricts the passage of this drug from the blood stream into the brain. Paroxetine O/W NEs for nose-to-brain targeting have been developed using the spontaneous emulsification method. Ex vivo studies carried out on porcine nasal mucosa revealed that permeation occurs with about a threefold enhancement in comparison with paroxetine suspensions. Forced swimming tests and locomotor activity tests were carried out as behavioral studies on Wistar rats to study the antidepressant effect of NEs. The treatment of depressed rats with nasal paroxetine NEs significantly improved their behavioral activities with respect to oral paroxetine suspensions. Histopathological studies were carried out on the brain tissues of Wistar rats, and brain tissues treated with paroxetine nasal NEs showed decreased degeneration and damage in the vesicular nuclei in comparison to the naïve group. The authors claimed that this decreased degeneration occurred because the nasal administration of paroxetine NE targeted the drug in an amount that was therapeutic to the brain.

Quetiapine, a dibenzothiazepine derivative, is an antipsychotic drug used for the treatment of schizophrenia, a psychotic disorder that affects about 1% people all over the world [70]. However, this drug has some limits in conventional, oral therapy: It has low solubility in water, and following oral administration shows low bioavailability (5–15%) and a strong first-pass effect. For all these reasons, O/W NEs were prepared to target the drug directly into the brain following nasal administration [70]. Tween 80 was used as surfactant. In vivo studies carried out on male Wistar rats following the nasal administration of NEs containing Transcutol P and propylene glycol showed shorter Tmax compared with that of intravenous administrations, as well as higher drug transport efficiency into the brain. Brain distribution studies demonstrated that quetiapine is transported directly into the brain after the nasal administration of quetiapine NEs. The authors concluded that NEs are a promising strategy for the brain-targeted delivery of quetiapine.

Another interesting, potential application of nose-to-brain NEs is in the therapy of migraine headaches, which are the most common neurological vascular headache disease, characterized by an intense and pulsating pain around the head [71]. Migraine headaches involve abnormal sensitivity of brain arteries that leads to rapid changes in the artery diameter and, as a result, to other arteries in the brain and scalp dilating and resulting in terrible pain in the head. Zolmitriptan is a drug used in the treatment of migraine headaches because it has a selective action on serotonin receptors and is effective in reducing symptoms [47]. Zolmitriptan is available commercially as an disintegrating oral tablet. The oral therapy has many drawbacks, however, such as first-pass metabolism and low bioavailability (40%), slow onset of action, a short half-life (1–2 h) and incomplete pain relief with the recurrence of headaches. In addition, during the migraine attack many patients suffer from nausea and/or vomiting that can make the oral treatment ineffective. Zolmitriptan mucoadhesive NEs were prepared and characterized in terms of zeta potential, particle size, morphology and ex vivo permeation through the nasal mucosa [47]. In vivo pharmacokinetics studies were carried out in mice in comparison with intravenous and nasal solutions of zolmitriptan. The addition to the NE of chitosan as a mucoadhesive agent in 0.3% enhanced the residence time and zeta potential of the formulation with no relevant effect on the globule size. In vivo studies carried out on Sprague–Dawley rats showed that the mucoadhesive (chitosan containing) NE presents higher AUC and shorter Tmax in the brain than intravenous or the nasal solutions. This behavior has been related to the presence of chitosan and higher permeability of the formulation. This study demonstrated that zolmitriptan mucoadhesive NEs are promising drug delivery systems to enhance efficacy in the treatment of migraine headaches.

The nose-to-brain passage of NEs has been studied through bioimaging by Ahmad et al. [72]. A series of near-infrared fluorescent diaza-indacene (BODIPY) and aza-BODIPY dyes were developed. Chitosan coated NEs (prepared with high-shear homogenizer) were labeled with these fluorescent probes to study the translocation profiles of integral NEs along the passages from the nose to the brain with imaging both in vivo and ex vivo using male SD rats. After administration, in vivo live imaging was achieved. Furthermore, at 1 h after NE administration the animals were sacrificed and the nasal mucosa, brain and trigeminal nerve were excised. The ex vivo imaging of the nasal mucosa, brain and trigeminal nerves were then taken and the brain was sliced. Particle size, rather than surface coating with chitosan or thickening with thermosensitive in situ gel, plays the most important role in determining the in vivo fate of NEs. NEs of about 100 nm have a prolonged residence duration in the nasal cavity, whereas NEs of larger sizes are characterized by faster clearance. Imaging showed that NEs of 100 nm were transported through the trigeminal and the olfactory nerves to the olfactory bulb. The transport of larger NEs along the nose-to-brain passage is lower and this can be due to the higher mucociliary clearance.

Recently, kaempferol-loaded NEs have been described for nose-to-brain targeting [73,74]. Kaempferol is a natural flavonol that can be found in different plant species. It presents anti-oxidant, anti-inflammatory, neuroprotective and anti-tumor properties that can be useful in the therapy of brain tumors such as gliomas [73]. Kaempferol-loaded NEs, with and without chitosan, were prepared by high pressure homogenization techniques and were characterized by their globule size, zeta potential, drug content, pH, viscosity, mucoadhesive strength and morphology. Results showed nanometric globule size, high loading capacity and entrapment efficiency, and NEs were able to preserve the antioxidant potential of kaempferol. Ex vivo diffusion studies were carried out using a Franz diffusion cell through a freshly isolated pig nasal mucosa and their activity was tested on C6 glioma cell lines. The NEs showed no toxicity towards nasal mucosa, and ex vivo permeation was higher in the formulations containing chitosan in respect to those without. A reduced C6 glioma cell viability through induction of apoptosis was obtained from NEs containing chitosan to a greater extent than either free drug solution or NE without chitosan. Furthermore, to evaluate the brain targeting after nasal administration, a preliminary brain distribution study of kaempferol was carried out in rats. The drug concentration (ng/g of tissue) in brain tissue after nasal administration of the NE formulation containing chitosan was found to be significantly higher with respect to NEs without chitosan or drug solutions.

One of the most recent papers published on the use of NEs for nose-to-brain delivery is by Haider et al., who prepared rivastigmine NEs for enhanced brain delivery through nasal administration [75]. Rivastigmine hydrochloride is a drug that has been proposed for the therapy of Alzheimer’s disease, as it helps in prevention of acetylcholine hydrolysis through the enzymes acetylcholinesterase and butyrylcholinesterase and thereby increases central cholinergic function and availability of acetylcholine. Box–Behnken design was used to statistically optimize the formulation parameters for the preparation of NEs. Ex vivo diffusion studies were carried out using Franz diffusion cells through freshly excised goat nasal mucosa. In vivo studies were carried out on Wistar rats. For the preparation of NEs, the selection of the oils led to the choice of Capmul MCM, whereas among surfactants Tween 80 and among co-surfactants Transcutol-P were chosen. Ex vivo diffusion studies done on goat nasal mucosa showed that the cumulative amount of drug permeation through nasal mucosa for NE was higher than that of the drug solution used as a comparison. In vivo studies showed that the brain drug concentration after nasal administration of NEs was significantly higher than that achieved after nasal administration of solutions and intravenous administration of NEs.

NEs loaded with chloramphenicol, based on palm kernel oil esters and suitable for meningitis treatment have been described [76,77], even if no specific indication about the possible nasal use of these formulations has been given. 

NEs containing amiloride have been prepared and administered intranasally in mice. Oleic acid, Tween 20 and Carbitol were employed as oil, surfactant and co-surfactant, respectively. For the emulsion preparation, a high-energy ultra-sonication method was used. An enhanced drug brain bioavailability was found as compared to intravenous administration [78].

Lastly, a paper by El-Zaafarany et al. describes lipid-based nanovectors defined as emulsomes and containing oxcarbazepine [79]. These dispersed systems can be considered the borderline between NEs and Solid Lipid Nanoparticles (SLNs). In fact, according to the authors, in the emulsomes the dispersed phase is constituted by “particles” comprising lipid cores stabilized by phospholipid layer(s) that surrounds the lipid core at the aqueous interface. The lipidic core of these particles is in a solid phase, rather than oil in a fluid phase. Oxcarbazepine is a drug used for the treatment of epilepsy. The aim of this work was the preparation of this lipidic nanocarrier aimed at a direct nose-to-brain targeting. Ex vivo studies carried out using rat nasal mucosa to check possible changes induced by emulsomes showed no histopathological alteration. Pharmacokinetic studies carried out in rats showed that oxcarbazepine-loaded emulsomes slowed drug elimination, and a high drug concentration was achieved in the blood stream for a longer time periods, as compared to the free drug administrations.

In Table 2 the schematic classification based on drug loaded in NEs is reported.

## 4. Conclusions

Nanoemulsions are formulations that are more and more important in the field of nanomedicine. Their characteristics (nanodroplets with a high surface) make them suitable for nose-to-brain delivery. Mucoadhesive polymers can be added in their composition to slow down nasal clearance. The presence of chitosan as an additional excipient plays a double role, because it is mucoadhesive and has penetration enhancing properties on nasal mucosa.

As shown by Table 2, there are many examples in the literature of recent years of nanoemulsion-loaded drugs with different therapeutic goals in brain diseases. The pathologies are all important and serious; many of these diseases, if not treated effectively, can reduce the quality of life or even lead to death. 

The nose-to-brain delivery is often an alternative to oral therapies for the CNS that can present problems, usually related to the characteristics of the drug. There are many reasons a CNS drug can be a good candidate for intranasal nanoemulsion, as an alternative to the oral administration (Table 1). 

However, as demonstrated by the reported literature, intranasal administrations of NEs often lead to better results, also with respect to intravenous administrations. These good results can be explained by mechanisms of transcytosis/endocytosis of the nanodroplets by the brain endothelial cells. Moreover, the surfactant(s) present in the nanoemulsions could have a fluidizing effect on endothelial cell membranes, determining an enhanced drug permeability and favoring by this mechanism the olfactory and trigeminal pathways.

Nanoemulsions for nasal administration represent a promising strategy for nose-to-brain drug delivery and to achieve CNS targeting for the treatment of neurodiseases. However, clinical studies of these formulations are still needed to demonstrate their appropriateness in clinical practice. 

Many efforts must be made to further improve the performances of nanoemulsions. Future perspectives could consider the use of additional excipients. For instance, it is known that cyclodextrins can be used for the preparation of formulations able to cross the BBB, administered through nasal or oral/parenteral routes [16,23,80,81]. New penetration enhancers and modulators of cytochrome P450 could be proposed to improve the selectivity and entity of penetration through the BBB [82,83,84].

## Figures and Tables

**Table 1:** Characteristics that make the central nervous system (CNS) drug a candidate for nasal NE, as an alternative to oral administrations.

Low capacity to cross the Blood-Brain-Barrier
Low solubility in water (and consequently poor bioavailability)
Low/irregular absorption (and consequently poor bioavailability)
Active substrate of intestinal P-glycoprotein
Problems of stability (pH, hydrolysis, oxidation, under gastro-intestinal conditions)
Intestinal metabolism (enzymatic degradation)
First-pass metabolism (enzymatic degradation)
Need to reduce dosage (i.e., in case of chronic therapy and/or to avoid side effects connected to the high oral dosage used for overcoming the Blood-Brain-Barrier)
Slow onset of action
Bitter/unpleasant taste of the drug

**Table 2:** Nanoemulsions designed for nose-to-brain delivery, and classification based on the drug.

Drug/Probe	Therapy of	Reference
Risperidone	Schizophrenia	[48]
Olanzapine	Schizophrenia	[49]
Ergoloid mesylate	Antiaging	[51]
Amiloride	Antiepileptic	[52]
Ziprasidone hydrochloride	Antipshychotic	[53]
Saquinavir mesylate	HIV infections	[28]
Nimodipine	Cerebrovascular spasm and senile dementia	[54]
Curcumin	Neurodegenerative diseases	[55]
Resveratrol	Parkinson’s disease	[58]
Curcumin/Resveratrol	Age-related neurodegenerative diseases	[60]
Cyclosporine-A	Neuro-protective	[61]
anti-TNFα siRNA	Neuro-inflammations	[62]
Riluzole	Amyotrophic lateral sclerosis (ALS)	[63]
Selegiline	Parkinson’s disease	[65]
Thymoquinone	Cerebral ischemia	[68]
Paroxetine	Depression and anxiety	[69]
Quetiapine	Schizophrenia	[70]
Zolmitriptan	Migraines	[47]
Aggregation-caused quenching (ACQ) probes	Labeling action	[72]
Kaempferol	Gliomas	[73,74]
Rivastigmine	Alzheimer’s disease	[75]
Chloramphenicol	Bacterial meningitis	[76]
Amiloride	Epilepsy	[78]
Oxcarbazepine	Epilepsy	[79]
